# Fruit Freshness Classification and Detection Based on the ResNet-101 Network and Non-Local Attention Mechanism

**DOI:** 10.3390/foods14111987

**Published:** 2025-06-04

**Authors:** Yuan Shu, Jipeng Zhang, Yihan Wang, Yangyang Wei

**Affiliations:** 1 Architecture and Design College, Nanchang University, Nanchang 330031, China; 2 School of Art, Wuhan Business University, Wuhan 430056, China

**Keywords:** ResNet-101, Non-local Attention, fruit freshness detection, deep learning, image processing, intelligent agriculture

## Abstract

Fruit freshness monitoring represents one of the key research foci in the quality control of fruits and vegetables. Traditional manual inspection methods are characterized by subjectivity and inefficiency, which renders them unsuitable for large-scale and real-time detection demands. Automated detection methods based on deep learning have increasingly attracted attention. In this study, a fruit freshness classification method based on the ResNet-101 network and a Non-local Attention mechanism is proposed. By embedding a Non-local Attention module into ResNet-101, subtle surface feature variations of the fruit are captured, thereby enhancing the model’s capacity to identify rotten areas and detect variations in color under complex backgrounds. Experimental results show that the improved model achieves a precision of 94.7%, a recall of 94.24%, and an F1-score of 94.24%, outperforming conventional ResNet-101, ResNet-50, and VGG-16 models. In particular, under complex environmental conditions, the model demonstrates significantly improved robustness in image processing. The combination of the Non-local Attention mechanism with the ResNet-101 model can substantially enhance the accuracy and stability of fruit freshness detection, which is applicable to real-time monitoring tasks in intelligent agriculture and smart logistics.

## 1. Introduction

Globalization has led to diversification in food demands around the world, and the production, sales, and consumption chains have become increasingly fragmented and intricate [1]. In the fruit and vegetable supply industry, rigorous sorting and grading of produce—including the removal of damaged, diseased, or substandard fruits—can effectively ensure the quality of market-bound products [2]. For example, different preservation techniques are employed for various fruits, such as the widespread application of chitosan for controlling weight loss in fresh strawberries (*Fragaria* × *ananassa*), raspberries (*Rubus idaeus*), mangoes (*Mangifera indica*), lychee, and blueberries [3], while papaya is preserved by reducing the respiration rate to delay the ripening process [4]. The ability to maintain optimal freshness during storage under limited time is a critical market requirement. Consequently, fruit freshness during circulation is directly linked to consumer health and purchasing behavior, rendering freshness detection a vital task in modern food quality control [5]. Due to environmental factors such as temperature, humidity, and illumination affecting fruits during storage, transportation, and sale, their freshness is in constant flux. Elevated temperatures accelerate the consumption of sugars, acids, and other nutrients in the fruit, leading to over-ripening, softening, and rotting. Furthermore, enzymatic reactions—such as the browning reaction induced by polyphenol oxidase—speed up, resulting in changes in color and deterioration of texture [6]. For instance, fresh peaches, apricots, and cherries may be preserved for approximately 4 months at −12 °C, about 18 months at −18 °C, and for over two years under freezing conditions with sugar treatment and storage at −24 °C [7]. Ultraviolet light can degrade light-sensitive compounds (such as chlorophyll and certain vitamins), causing fading of fruit color and loss of nutritional components (e.g., vitamin C, vitamin A) [8]. In recent years, research on the microbial safety and quality control of fruits has increased. The freshness of fruits and vegetables is highly associated with their microbial safety; during harvesting, transportation, and storage, fruits are prone to microbial contamination which accelerates spoilage [9]. Traditional manual inspection methods primarily rely on visual assessment and simple instrumentation (e.g., colorimeters and hardness testers) for quality evaluation, but these approaches capture only a subset of the parameters affected by microbial invasion or intrinsic metabolic changes [10]. Although some laboratories employ microbial culturing and chemical analysis methods—which yield accurate results—the prolonged detection cycle and high cost render them unsuitable for real-time monitoring [11]. Therefore, the development of an automated, real-time, and accurate detection technology for assessing fruit freshness holds significant theoretical and practical value.

Deep learning technologies, especially convolutional neural networks (CNNs), have achieved remarkable success in image classification and object detection tasks, and have demonstrated extensive application prospects in fruit detection. For instance, Valentino et al. designed a CNN-based fruit freshness detection system using a Kaggle dataset to classify fruits as fresh or rotten, implementing real-time detection via a web application to offer an efficient, automated solution for the fruit supply chain [12]. Similarly, Pathak and Makwana proposed a CNN-based model for automatically classifying fruit as “fresh” or “rotten”, achieving a classification accuracy of 98.23% through hyperparameter optimization and the integration of multiple convolutional and pooling layers [13]. Gao et al. employed data augmentation to expand 800 images to 12,800, achieving efficient detection of fruits in dense canopies with a mean average precision (mAP) of 87.9%, thereby supporting robotic harvesting path planning and collision avoidance [14]. Apolo-Apolo et al. developed an unmanned aerial vehicle (UAV)-based citrus fruit monitoring system combined with deep learning, which significantly improved the accuracy of fruit size estimation and yield prediction [15]. Amin et al. proposed a transfer-learning-based CNN method using the AlexNet model to efficiently classify fruits as fresh or rotten, achieving nearly 100% accuracy on multiple public datasets, thus offering an automated solution for the fruit processing industry [16].

Although machine learning algorithms based on color, shape, and texture features can perform well in controlled scenarios, they exhibit limited representation ability in complex environments—where factors such as lighting variations, occlusions (e.g., by leaves or branches), and fruit density undermine detection accuracy. Even though deep learning models exhibit excellent performance in fruit detection, they are highly dependent on large-scale, high-quality annotated datasets. The acquisition and annotation of such datasets are time-consuming and labor-intensive tasks, particularly in complex environments where high annotation precision is required [17]. Hence, there is a need to improve models to better cope with complex scenarios.

The self-attention mechanism is a powerful tool for capturing dependencies among different positions of the input data and has been widely applied in natural language processing (NLP) and computer vision [18]. For instance, Lin et al. proposed a transfer-learning- and attention-mechanism-based CBAM-SEResNet-50 model for sweetener identification, which achieved a feature extraction accuracy of 95.6% by combining Squeeze-and-Excitation (SE) module and a Convolutional Block Attention Module (CBAM) [19]. In maize leaf disease detection, Qian et al. introduced a self-attention mechanism that significantly improved the model’s ability to focus on subtle lesions in complex backgrounds, effectively suppressing background noise and further enhancing classification accuracy [20]. The Non-local Attention mechanism, which is able to capture relationships between any two pixels in an image through global modeling, overcomes the limitations of conventional CNNs that rely solely on local receptive fields and enhances sensitivity toward subtle changes. Chen et al. proposed an image compression method that integrates the Non-local Attention mechanism and context optimization, achieving compression efficiencies that surpass those of conventional and mainstream deep learning methods [21]. Huang et al. introduced a TransFuser model based on Non-local Attention for fusing key features and retinal images, achieving significantly superior performance compared to existing models on multiple evaluation metrics [22]. In the context of fruit freshness detection, the incorporation of Non-local Attention enables the model to better recognize subtle damage such as slight rotting areas or color discrepancies, thereby enhancing both classification accuracy and robustness [23].

This study proposes a novel fruit freshness classification model that fuses ResNet-101 with a Non-local Attention mechanism. By embedding non-local modules into the deep convolutional layer 4 (Conv4) and convolutional layer 5 (Conv5) stages of Residual Network-101 (ResNet-101), the model more effectively captures subtle features—such as localized decay and color fading—thereby markedly enhancing robustness and classification accuracy under complex environmental conditions. To improve generalization and practical applicability, we construct a comprehensive dataset that combines publicly available images with photographs acquired in realistic settings featuring diverse illumination, occlusion, and background scenarios, encompassing multiple fruit types at varying freshness levels. The experimental protocol incorporates a systematic preprocessing pipeline and class-balancing strategies and evaluates performance using multiple metrics—precision, recall, and F1-score—followed by comparative validation against baseline networks. Leveraging Gradient-weighted Class Activation Mapping (Grad-CAM) for visualization, we further provide an interpretability analysis at the attention-region level, confirming that the Non-local Neural Network Attention (Non-local Attention) mechanism strengthens the model’s ability to identify critical decay regions. The preliminary assessment of deployment on edge devices indicates that, despite a modest increase in parameter count and inference latency, the overall computational overhead remains acceptable. This study supports real-time implementation in smart agriculture and intelligent logistics scenarios, enabling timely decision making and efficient resource management.

## 2. Materials and Methods

### 2.1. Dataset Construction and Preprocessing

In the task of fruit freshness detection, the quality and diversity of the dataset are decisive factors in model performance. In this study, a comprehensive dataset—combining publicly available datasets and self-collected images—was constructed to ensure the inclusion of various fruit types and their freshness levels, thereby enhancing the model’s generalization capability.

The study utilized a public fruit image dataset from Kaggle (www.kaggle.com), which contains an extensive collection of fruit categories and samples. It should be noted that the diversity in terms of acquisition time (season or period), location, and weather conditions was not systematically designed or experimentally validated in this dataset. For the experiments on freshness detection, apples, bananas, and oranges were selected.

**Figure 1:**
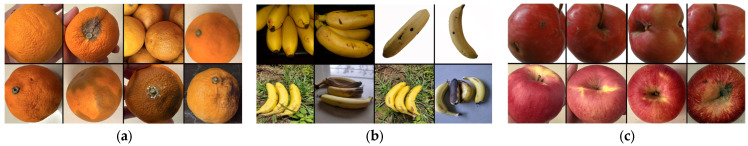
Selected sample images: (**a**) orange; (**b**) banana; (**c**) apple.

To improve the diversity and authenticity of the dataset, additional fruit images were captured under real-world conditions, including variations in illumination, complex backgrounds, and a distribution of different freshness states. The images were captured using high-resolution smartphones, and the fruit types were kept consistent with those from the public dataset. Figure 1 shows sample images from the complete dataset.

#### 2.1.1. Data Distribution

To ensure that the model could accurately learn the features corresponding to different freshness levels, the images for each fruit type were annotated according to two freshness levels [24]. The images were labeled as described in Table 1.

#### 2.1.2. Data Preprocessing

**Figure 2:**
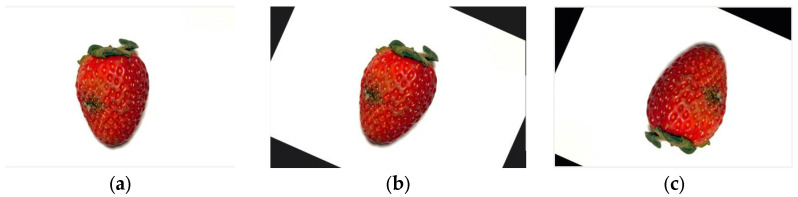
Image Augmentation: (**a**) original image; (**b**) rotation; (**c**) flipping.

To enhance the model’s robustness and generalization ability, all images in the dataset were resized to 224 × 224 pixels to conform to the input specifications of mainstream CNN models. Pixel values were normalized to the range [0, 1] to facilitate gradient optimization. Geometric transformations, such as random rotation (±30°), horizontal and vertical flipping, and random cropping, were applied to augment the dataset and improve the model’s adaptability to images from different viewpoints [25]. Brightness and contrast adjustments were also performed to simulate images under varying lighting conditions, as demonstrated in Figure 2. Finally, slight Gaussian noise was added to some images to enhance the model’s robustness against interference. For certain fruits with fixed orientation or distinctive shapes (e.g., bananas), some bias may be introduced. Table 2 presents the contribution of data augmentation techniques to the number of samples.

Owing to the imbalance in the number of samples across different freshness levels, oversampling and weighting adjustments were adopted to balance the class distribution. For the rotten category with fewer samples, data augmentation and sample duplication were employed to mitigate model bias. The dynamic characteristics of freshness variations differ among fruit types, and the feature representation for the rotten category is more complex due to limited sample availability, which may affect the model’s performance on the “bad” category.

### 2.2. Network Architecture Design

#### 2.2.1. Core Architecture of ResNet-101

ResNet is a deep convolutional neural network introduced by He et al., its core concept is to use residual learning to mitigate the vanishing gradient and degradation problems in deep networks [26]. As network depth increases, traditional CNNs suffer from declining performance due to the vanishing gradients; ResNet alleviates this problem by introducing skip connections that allow information to bypass several layers.

ResNet-101 is one of the deeper networks in the ResNet series, composed of 104 convolutional layers and a total of 33 blocks. Out of these, 29 blocks directly use the output of the preceding block (via residual connections) for summation at the end of each block, which then serves as the input for subsequent blocks [27]. The remaining 4 blocks obtain the output from the preceding block, apply a 1 × 1 convolution with a stride of 1 followed by batch normalization, and then perform the summation operation. Compared to traditional machine learning methods (e.g., Support Vector Machine, Artificial Neural Network), this design yields a higher classification accuracy [28].

(1) $$Y=F\left(x\right)+x$$

where $\;\;F\left(x\right)$ represents the convolution operation and x is the input. This design converts the optimization of a deep network into the optimization of residuals, thereby enhancing training efficiency. ResNet-101 employs Bottleneck Blocks to reduce computational cost and the number of parameters [29]. The Bottleneck Blocks comprise a 1 × 1 convolution for dimensionality reduction, a 3 × 3 convolution for feature extraction to capture local features, and another 1 × 1 convolution for dimensionality expansion to restore the channel count. This design reduces the parameter count while preserving the expressive capability of the network. Skip connections refer to the direct summation of the input, denoted as x, with the convolutional output $F\left(x\right)$ within each residual unit, thereby ensuring that the input features are transmitted to deeper layers without any loss. When the dimensions of the input and output differ, a 1 × 1 convolutional projection is employed to align the dimensions. The architecture is detailed in Table 3.

ResNet-101 demonstrates excellent performance in complex visual tasks owing to its deep architecture, which enables the network to learn abstract and fine-grained feature representations, thereby enhancing the sensitivity to subtle differences (e.g., slight rotten areas or color changes on the fruit surface). For the task of fruit freshness classification, detailed features often contain crucial discriminative information, and a deeper network can effectively merge low-level and high-level information. Although lightweight network architectures such as Mobile Convolutional Neural Network (MobileNet) and ShuffleNet Convolutional Neural Network (ShuffleNet) offer reduced parameters and computational complexity suitable for real-time applications [30,31]; the current task requires detailed feature extraction, and these lighter models tend to have limitations in depth and expressive power. In contrast, ResNet-101 captures complex features at multiple scales, achieving effective fusion between low-level and high-level representations [32] and demonstrating greater advantages in maintaining high classification accuracy and robustness.

#### 2.2.2. The Non-Local Attention Mechanism

Non-local Attention is a mechanism designed to overcome the limitations of local feature extraction by modeling global context, which captures long-range dependencies between any two positions in an image, thereby enhancing the network’s sensitivity to subtle changes and important regions [33]. This global contextual capability is particularly advantageous for fruit freshness detection, where the model must accurately focus on regions such as localized rotting, fine cracks, or fading color. Modules such as the Squeeze-and-Excitation Block (SE-Block), primarily focusing on adaptive recalibration in the channel dimension, and Convolutional Block Attention Module (CBAM), which introduces spatial attention along with channel information [34,35], are commonly used; however, the Non-local Attention module computes similarity between any two spatial positions through global self-attention and constructs a global attention weight map [28]. By integrating global information from the entire feature map, it effectively alleviates the limitations of conventional CNNs that rely solely on local receptive fields, enhancing the perception of subtle changes.

The core of the Non-local Attention mechanism is to compute the correlation between any two positions in a feature map and to reallocate attention weights accordingly. Suppose the input feature map X ∈ $R^{C\times H\times W}$, where C, H, and W represent the number of channels, height, and width, respectively; thus, the main computation process is presented here.

(a) Feature Mapping and Correlation Computation:

The input feature map X is transformed via two distinct linear transformations to generate two feature maps, $f\left(X\right)$ and $g\left(X\right)$, which correspond to the query and key features, respectively. Then, the dot–product correlation between $f\left(X\right)$ and $g\left(X\right)$ is computed to obtain a global attention matrix:

(2) $$S\left(i,j\right)={f\left(X_{i}\right)}^{T}g\left(X_{j}\right)$$

In this context, S(i,j) denotes the correlation between positions i and j within the feature map.

(b) Generation of Attention Weights:

The correlation matrix S is normalized via the Softmax function to obtain the attention weight matrix A, whereby the weight at each position indicates the degree of dependency on other positions.

(3) $$A(i,j)=\frac{exp\left(S(i,j)\right)}{\sum_{j=1}^{N}exp\left(S(i,j)\right)}$$

In this context, N = H × W. N is the total number of spatial positions.

(c) Feature Enhancement:

The input feature map X undergoes an additional linear transformation to produce the value feature $h\left(X\right)$.

The value feature $h\left(X\right)$ is aggregated through a weighted summation using the attention weights *A*, thereby generating an enhanced feature map:

(4) $$Z_{i}=\sum_{j=1}^{N}A(i,j)h\left(X_{j}\right)$$

(d) The enhanced feature map Z is combined with the input feature map X via a residual connection to obtain the output feature map Y.

(5) Y=Z+X

In this study, the Non-local Attention module is embedded into the Conv4 and Conv5 layers of the ResNet-101 network, as these layers generally contain more abstract information such as the shapes, textures, and structural relationships of objects. Conv4, which contains 23 residual units, is primarily responsible for extracting mid-to-high-level features. The addition of Non-local Attention significantly enhances the model’s capacity to perceive complex features. For the high-level feature extraction layer Conv5, the inclusion of Non-local Attention supports the classification task by enriching the global contextual information. Early layers (e.g., Conv1 or Conv2), which predominantly extract low-level features (such as edges, corners, and local textures) with high locality and redundancy, may not benefit from global context integration and might even introduce instability due to noise in low-level features [36]. Preliminary experiments embedding the Non-local Attention module in the early layers revealed no significant improvements in final classification accuracy or model convergence speed; in some cases, performance slightly deteriorated. This is primarily attributed to the localized nature of early features, which do not gain significantly from global contextual integration. In contrast, embedding in Conv4 and Conv5—where the spatial dimensions of the feature maps have already been reduced—keeps the computational overhead in check while ensuring performance gains.

By employing this module embedding strategy, the Non-local Attention mechanism is efficiently integrated with the residual learning framework of ResNet-101, thereby enhancing feature extraction while preserving training efficiency.

### 2.3. Experimental Design and Evaluation

**Figure 3:**
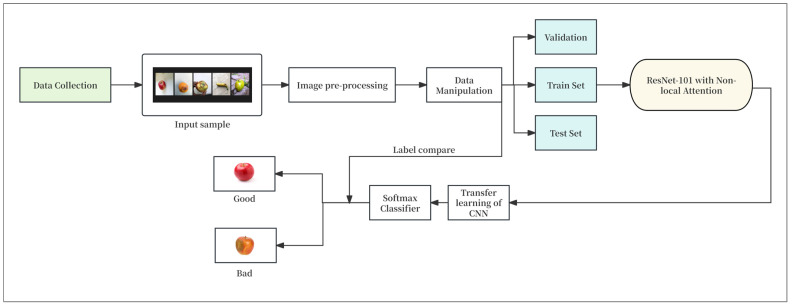
Experimental workflow.

To validate the effectiveness of the proposed method combining ResNet-101 and the Non-local Attention module for fruit freshness detection, a systematic experimental protocol was designed. The experimental setup included the model training process, the selection of evaluation metrics, and the configuration of the experimental environment to comprehensively assess the performance in terms of accuracy, generalization capability, and time efficiency, as depicted in Figure 3.

#### 2.3.1. Model Training

The publicly available dataset and the self-collected dataset described earlier were used as training data. Preprocessing steps (such as image scaling, normalization, and augmentation) were applied to ensure that the model was exposed to diverse input conditions. Class balancing strategies were adopted to mitigate training biases due to class imbalance.

The cross-entropy loss function was employed for training, as it effectively optimizes the class probability distribution in classification tasks:

(6) $$L_{CE}=-\sum_{i=1}^{N}Y_{i}\log\left({\hat{Y}}_{i}\right)$$

**Figure 4:**
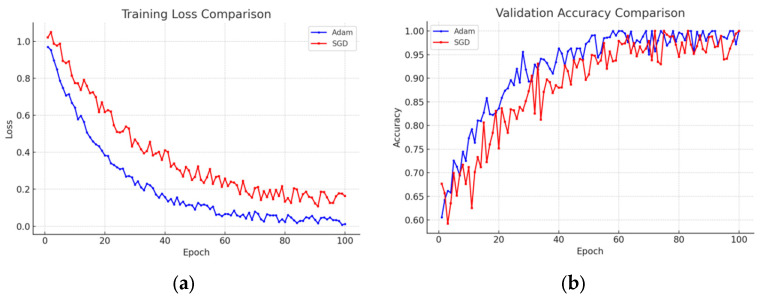
Validation accuracy comparison: (**a**) training loss comparison; (**b**) training accuracy comparison.

In this context, $Y_{i}$ represents the actual category labels, whereas ${\hat{Y}}_{i}$ denotes the predicted category probabilities. Regarding the optimizer, the Adam optimizer is employed; it combines the advantages of momentum and an adaptive learning rate, which accelerates convergence and effectively mitigates the issues associated with local minima [37]. Under identical hyperparameter settings, Adam’s adaptive learning rate adjustment mechanism enables it to achieve lower loss values with fewer iterations. Additionally, by integrating momentum along with adaptive estimates of the first and second moments, Adam ensures a smoother training process that is less susceptible to becoming trapped in local optima or entering oscillatory states. In contrast, Stochastic Gradient Descent (SGD), whether used with or without momentum, tends to be more sensitive to the learning rate, often necessitating more precise hyperparameter tuning and exhibiting slower convergence. The Adam optimizer has demonstrated robust performance across diverse tasks, with its default parameters ($\beta_{1}$, $\beta_{2}$, etc.) frequently yielding satisfactory outcomes, thereby reducing the hyperparameter tuning workload. On the other hand, SGD typically requires detailed, task-specific tuning—including setting the initial learning rate, determining the momentum factor, and devising a learning rate decay strategy—which increases the complexity and debugging time in practical engineering implementations [38]. In some experiments, both Adam and SGD were employed, and the training loss descent curves as well as the validation accuracy were recorded. As illustrated in Figure 4, the model optimized with Adam was able to rapidly reduce the loss during the initial training stages and achieve higher validation accuracy in a shorter period; whereas the model optimized with SGD, despite achieving satisfactory performance after extensive adjustments in later stages, exhibited a significantly longer overall training time and considerable fluctuations in the early phases, making it more prone to noise interference.

**Figure 5:**
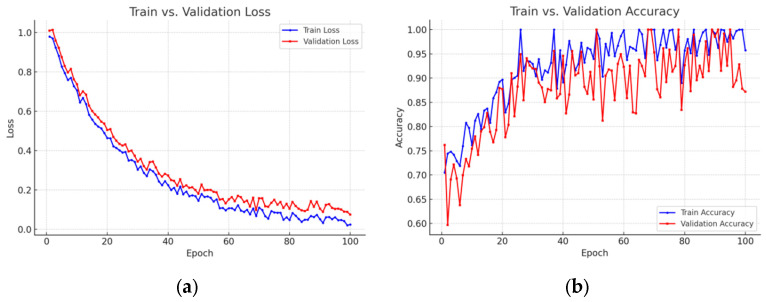
Train vs. validation: (**a**) loss comparison; (**b**) accuracy comparison.

The initial learning rate was set to 1 × 10^−4^ and dynamically adjusted based on model performance. A batch size of 32 was adopted to effectively utilize Graphics Processing Unit (GPU) parallelism without exceeding memory limits. The model was trained for 100 epochs, with performance evaluated after each epoch using the validation set. Figure 5 indicated that, after approximately 60–70 epochs, the training loss continued to decrease while the validation loss plateaued or slightly increased; meanwhile, the training accuracy kept rising even as validation accuracy improvements slowed or slightly declined. This divergence indicates that the model begins to overfit to noisy patterns in the training data [39].

To mitigate overfitting, an early-stopping strategy was adopted [40]. A patience window was defined (e.g., 5–10 epochs with no significant improvement in validation metrics), after which training was terminated. With early stopping enabled, the model maintained stable or slightly improved performance on the validation set, thereby reducing the risk of performance degradation under real-world conditions. The learning rate decay was implemented via cosine annealing [41] to facilitate fine-tuned model adjustments over the training period. Table 4 provides a detailed overview of the hyperparameters used during the training process.

#### 2.3.2. Evaluation Metrics

To comprehensively evaluate model performance, several widely used classification metrics were employed. Accuracy, defined as the proportion of correct predictions relative to the total number of samples, is calculated as follows:

(7) $$Accuracy=\frac{TP+TN}{TP+TN+FP+FN}$$

where TP is true positives, TN is true negatives, FP is false positives, and FN is false negatives.

Recall measures the model’s ability to capture target classes—particularly crucial for classes with poor freshness:

(8) $$Recall=\frac{TP}{TP+FN}$$

Precision quantifies the proportion of correctly predicted positive samples among all positive predictions:

(9) $$Precision=\frac{TP}{TP+FP}$$

The F1-score, defined as the harmonic mean of precision and recall, balances the trade-off between the two [42]:

(10) $$F1=2\times \frac{Precision\times Recall}{Precision+Recall}$$

To further validate the model’s generalization capability, k-fold cross-validation (with k = 5) was employed [43]. The dataset was partitioned into 5 subsets, and the model was trained and validated 5 times, each time using a different subset for validation. The average of these results was computed to reduce overfitting risks associated with any single split.

All experiments were conducted on an NVIDIA GTX4060 to accelerate model training and inference. The specific hardware configuration is provided in Table 5.

The deep learning framework employed was PyTorch 1.9.1, using CUDA 12.2 to accelerate computations.

#### 2.3.3. Experimental Settings

As shown in Table 6, to ensure robust training and generalization, 80% of the data was used for training and 20% was used for validation. In addition, the inference time of ResNet-101 during the testing phase was evaluated, comparing it against traditional models such as Visual Geometry Group-16 (VGG-16) and ResNet-50. This evaluation aimed to reveal the computational overheads in real-world deployment scenarios, particularly for edge devices.

## 3. Results

### 3.1. Comparative Analysis of Model Performance

**Figure 6:**
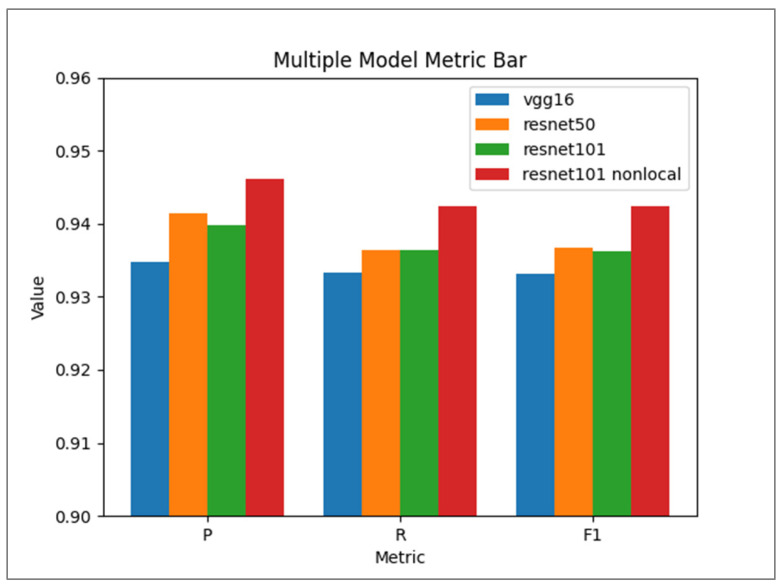
Performance comparison of ResNet-101 with Non-local Attention versus other models (where P is precision, R is recall, F1 is F1-score).

To comprehensively evaluate the efficacy of the improved model, extensive comparative experiments were conducted between the ResNet-101 model integrated with Non-local Attention and the conventional ResNet-101, alongside comparisons with (VGG-16) and ResNet-50 models. All models were trained and tested on the same dataset to ensure fairness and reliability. Precision, recall, and F1-score were used as evaluation metrics, as shown in Figure 6.

The ResNet-101 model with Non-local Attention achieved a precision of 94.7%, higher than that of ResNet-101 (93.98%), VGG-16 (93.47%), and ResNet-50 (94.15%). This improvement indicates that the model is more effective in identifying the true target classes in the fruit freshness classification task, reducing the occurrence of misclassifications. In terms of recall, the improved model reached 94.24%, surpassing ResNet-101 (93.64%), VGG-16 (93.33%), and ResNet-50 (93.64%). The elevated recall suggests better detection of true positive freshness levels, reducing misclassification as negatives. The F1-score of the improved model was 94.24%, which is superior to that of ResNet-101 (93.62%), VGG-16 (93.32%), and ResNet-50 (93.68%). These performance improvements can be attributed to the introduction of Non-local Attention modules at strategically selected stages. Preliminary experimental results show that when the model is trained using only the original dataset, it achieves a precision of 89.3%, a recall of 87.9%, and an F1-score of 88.1%. The application of data augmentation techniques significantly enhances the model’s generalization ability and robustness.

**Figure 7:**
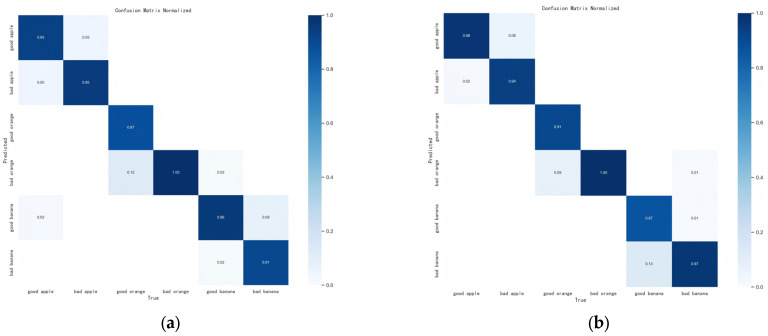
Comparison of confusion matrices: (**a**) ResNet-101; (**b**) ResNet-101 with Non-local Attention.

To further analyze the performance across different freshness categories, confusion matrices were compared. The confusion matrices provide a visual representation of the distribution of true positives and misclassifications (false positives and false negatives) in each class [44], which is instrumental in identifying the model’s weaknesses and guiding subsequent improvements. Figure 7 presents a comparison between the confusion matrices of the conventional ResNet-101 and the ResNet-101 combined with Non-local Attention. For the ResNet-101 model, misclassifications primarily occur in fruits with more apparent surface damage, such as those prone to discoloration or softening. In particular, bananas, whose ripening process is relatively subtle, are more prone to misclassification compared to other fruits. After integrating the Non-local Attention mechanism, the model’s accuracy in identifying “bad bananas” increased from 0.91 to 0.97. However, this improvement in sensitivity to subtle features led to a decrease in the model’s accuracy for recognizing “good bananas”.

### 3.2. Visualization and Comparative Evaluation of Feature Extraction

**Figure 8:**
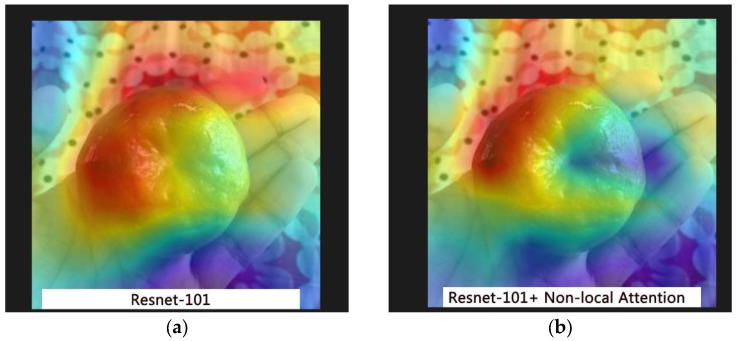
Grad-CAM visualization comparison: (**a**) ResNet-101; (**b**) ResNet-101 with Non-local Attention.

Grad-CAM is used to visualize the regions that the ResNet-101 and the Non-local Attention enhanced model focused on during prediction. Grad-CAM leverages the gradient information from the final convolutional layers to generate heat maps that indicate the regions influential for the final prediction [45], as illustrated in Figure 8. Warmer colors such as red and yellow indicate areas of higher relevance, where the model is strongly focused when making predictions, typically corresponding to regions with rotting, discoloration, or structural damage. Conversely, cooler colors such as blue and green represent areas with lower attention weights, which contribute less to the model’s decision.

The comparison shows that, after the integration of Non-local Attention, the model exhibits a more concentrated focus on the rotted regions, as well as improved detection of damage on the fruit surface.

In order to quantitatively assess the coverage of the attention regions, Table 7 defines the overlap rate (OR) relative to manually annotated rotten regions [46]. With the incorporation of the Non-local Attention mechanism, the overlap rate increased by approximately 8.2%, and the false detection rate was reduced by half, thereby enhancing the model’s practical utility.

Although the Non-local Attention mechanism contributes to performance improvement, it also increases the overall parameter count and FLOPs (Floating Point Operations) during inference [47]. Table 8 compares the training time, inference time, and number of parameters for various models. The improved model’s parameter count increased by approximately 2.3 million (M), and its inference time was only 1.2 millisecond (ms) longer than that of the conventional ResNet-101. Combined with its superior classification performance and modest time overhead, the enhanced ResNet-101 model remains practical for deployment.

**Figure 9:**
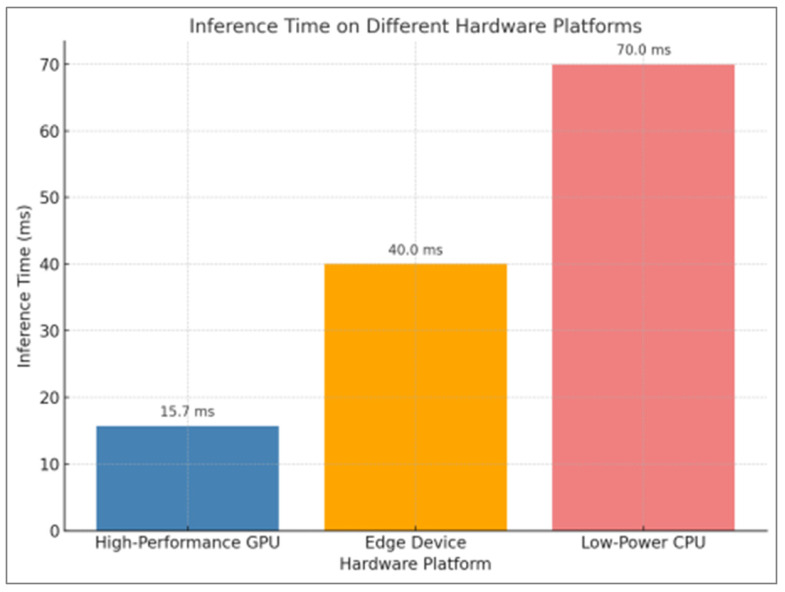
Inference time comparison on different hardware platforms.

For edge devices or embedded systems (such as Acorn Reduced Instruction Set Computing Machine-based mobile devices or smart cameras), hardware limitations (e.g., computational capacity and memory bandwidth) imply that additional computational overheads may have a more pronounced effect on inference time. Although large-scale tests were not conducted on every edge platform, preliminary simulations and experiments on low-power GPUs (Figure 9) suggest that—through model quantization, pruning, and optimizations (e.g., using TensorRT Optimization Library or Open Visual Inference and Neural Network Optimization)—the enhanced model remains feasible for deployment, with inference times within acceptable limits.

### 3.3. Robustness and Generalization Testing

**Figure 10:**
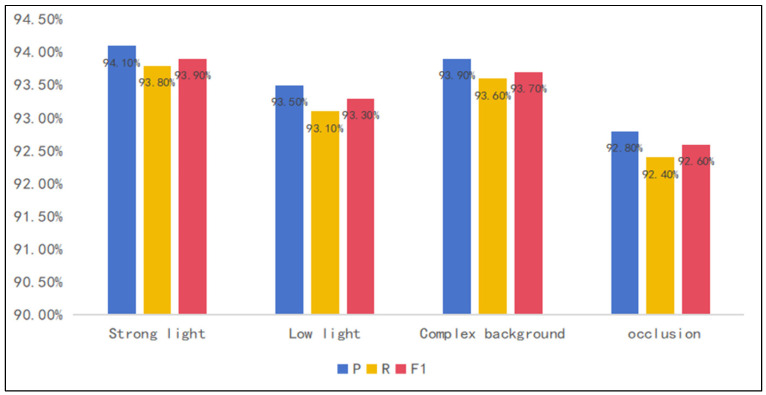
Model performance under different scenarios.

To validate the robustness and generalization of the improved model under complex conditions, an additional test set was acquired, comprising varied illumination conditions (strong light, dim light, shadows), different backgrounds (monochrome, complex), and occlusions (partial obstruction by leaves or other fruits). As shown in Figure 10, the model with Non-local Attention maintained a performance level exceeding 92% across these varied scenarios, demonstrating strong robustness and potential for practical applications.

## 4. Discussion

### 4.1. Data Scale and Diversity

Given that the overall sample size in the experiments was relatively limited, and that fruits in real circulation are subject to a myriad of factors such as geographical location, season, storage methods, and microbial contamination, leading to even more complex patterns of freshness variation, an increased dataset size and diversity is warranted. While the classification accuracy for specific fruits (e.g., apples) was high, the recall rate for some categories of other fruits (e.g., bananas) slightly declined. Furthermore, the present study focused primarily on fruits, yet vegetables which exhibit distinct freshness deterioration patterns (such as color fading and wilting for leafy vegetables, or skin wrinkling and moisture loss for root vegetables) were not deeply examined. Prior studies (e.g., Amin, Umer et al., 2023) have encompassed both fruits and vegetables with extremely high accuracy [16]. In future work, expanding the dataset for both fruits and vegetables to include a wider variety of species and extreme storage and transportation scenarios, as well as increasing the diversity of image samples (e.g., lighting conditions, humidity, temperature), will help significantly enhance the model’s adaptability and robustness.

### 4.2. Computational Complexity of the Non-Local Attention Mechanism and Its Practical Deployment

**Figure 11:**
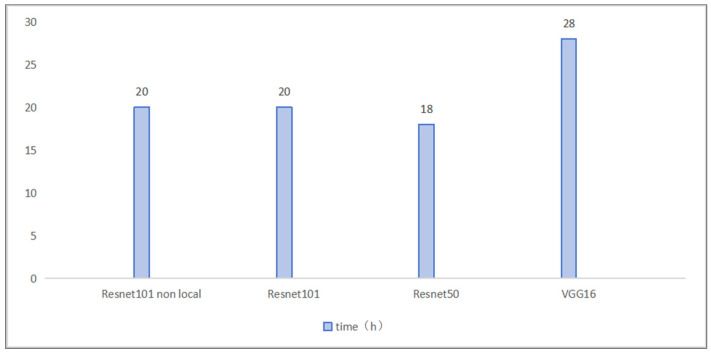
Training time comparison.

The integration of the Non-local Attention module into the ResNet-101 network enables a more precise capture of subtle fruit surface features (such as rotted areas and fading color), with significant improvements in accuracy, recall, and F1-score compared to the baseline. Although the integration slightly increases the number of parameters and marginally prolongs both training and inference times, it demonstrates good applicability for real-time applications. In environments with complex backgrounds, the module markedly enhances the model’s ability to focus on subtle rotted regions. However, the additional computational and memory overhead introduced by Non-local Attention poses challenges in scenarios with stringent real-time requirements and limited device performance. In the previous comparison of the model with the integrated Non-local Attention mechanism and the other three models, a comparative experiment was also conducted to evaluate the training times of these four models. Figure 11 illustrates the computational complexity of the baseline model for comparison. The training time for the ResNet-101 model with Non-local Attention was approximately 20 h, comparable to that of the standard ResNet-101, while the lighter ResNet-50 required nearly 18 h and VGG-16 required approximately 28 h. In high-throughput applications such as warehouse sorting and logistics transportation, deploying models with high computational loads may be problematic. Therefore, techniques such as model quantization, pruning, and knowledge distillation [48,49] should be considered to compress the model for efficient deployment on embedded platforms. In addition, distributed computing platforms, GPU acceleration, or specialized inference chips may be utilized to reduce latency per inference, ensuring the system can operate in environments with high throughput.

The increase in training time is primarily attributed to the heightened complexity of the model. With the integration of the Non-local Attention mechanism, the model is able to more accurately capture subtle feature variations, thereby improving classification accuracy and recall. Despite the increased computational cost during the training phase, the performance of the trained model during the inference phase still meets the requirements for real-time applications. The architectural choice of embedding global attention mechanisms only in deep feature layers represents a pragmatic compromise between computational complexity and accuracy. This selective enhancement ensures that the model remains feasible for real-time deployment.

To ensure continued model adaptability, the establishment of continuous monitoring and data collection mechanisms is recommended in future. Methods such as online learning or periodic retraining can facilitate dynamic parameter updating, allowing the model to adapt to evolving conditions [50]. The introduction of anomaly detection modules will help rapidly identify performance degradation and enable swift corrective measures [51]. Redundancy strategies such as deploying primary and backup detection units and fault-tolerant mechanisms can ensure the continuity of critical detection tasks in the event of hardware failure, network delay, or data noise [52]. Moreover, logging and real-time monitoring are essential for rapid fault localization and repair [53,54].

### 4.3. Subjectivity in Class Annotation and Freshness Definition

In this study, fruit samples were categorized into “good” and “bad” based on subjective criteria such as appearance, color, and the presence of mold or rotting regions. Although standardized criteria were employed during annotation, inconsistencies and bias are inevitable for samples on the borderline. Comparisons among different fruit types revealed that while some (e.g., apples and oranges) exhibit clearer surface characteristics, others (e.g., bananas) show greater ambiguity, underscoring the need for more refined and uniform annotation standards. Relying solely on external features may not fully capture the internal quality of fruits or vegetables. Future work could integrate objective physicochemical indicators (such as sugar–acid ratio, firmness, microbial load) or employ multimodal sensors to collect richer datasets, thereby enabling more objective and fine-grained categorization and enhancing classification reliability.

### 4.4. Robustness and Generalization in Complex Scenarios

Although the self-collected data attempted to simulate varied lighting conditions and complex backgrounds, the experiments were primarily conducted under relatively controlled conditions. Performance analysis across different scenarios showed that, under extreme conditions (e.g., strong direct light or complete occlusion), the model’s robustness decreased. While the performance tended to improve with a higher proportion of complex scenarios in the training set, discrimination between samples in the “bad” category still requires further refinement. In real-world circulation, fruits and vegetables may experience stacking, occlusion, deformation, as well as fluctuations in temperature and humidity, making surface features harder to capture accurately. Grad-CAM visualizations indicate that the Non-local Attention mechanism effectively enhances the focus on rotted areas; however, the model’s stability in large-scale, interference-rich settings must be further validated. Future investigations could incorporate data from additional modalities such as infrared and depth imaging to further improve detection accuracy and generalization in complex scenarios [55]. Fusion technologies integrating temperature, humidity, and spectral data could also enhance the evaluation accuracy of fruit and vegetable quality [56].

### 4.5. Model Interpretability and Broader Application Scenarios

While Grad-CAM offers insights into the regions on which the model focuses, the interpretability of deep learning models in their decision-making process remains limited. Comparative heat maps indicate that the model accurately captures key features such as rotted regions and color discrepancies. Nevertheless, the attention distribution in borderline cases appears uneven, warranting further refinement of the attention mechanisms. In applications related to food safety and logistics efficiency, stakeholders often require transparent explanations of the model’s decision-making process to enable traceability in the event of disputes or misclassifications. Freshness detection for fruits and vegetables represents only one aspect of food quality evaluation; incorporating multimodal data such as spectral imaging, gas sensing, or microbial detection could provide a more comprehensive quality control solution for intelligent agriculture and smart logistics [57]. By jointly analyzing detection results, environmental conditions, and logistics data on a cloud platform, managers could obtain full-process and comprehensive quality monitoring and early warning information to guide storage, transport scheduling, and market segmentation. Data integration via cloud platforms and big data analytics would further enhance the scientific accuracy and reliability of decision making [58].

## 5. Conclusions

This study focuses on the problem of fruit freshness classification and detection based on advanced image processing techniques, aiming to improve the accuracy and robustness of freshness assessment through deep learning models. By integrating the ResNet-101 network with a Non-local Attention mechanism, an optimized image processing approach was devised to enhance fruit freshness detection performance. Experimental results indicate that the improved model outperforms the conventional ResNet-101 as well as other common deep learning networks like VGG-16 and ResNet-50 across multiple evaluation metrics. In particular, under complex conditions (such as varied lighting, complex backgrounds, and occlusion), the model incorporating Non-local Attention demonstrates superior robustness. Although the inference time is slightly increased, it remains within an acceptable range for real-time applications on edge devices and large-scale deployments. The inclusion of the Non-local Attention mechanism in the network architecture significantly improves the model’s capacity to capture subtle rotted areas and color discrepancies on fruit surfaces. Comprehensive data collection, augmentation strategies, and visualization techniques provide a broader foundation for error analysis and further model refinement. With these improvements, the model holds promise for offering more precise and reliable fruit freshness monitoring solutions in intelligent agriculture and smart logistics.

Future research will focus on further optimizing the model’s lightweight design to enhance real-time performance and computational efficiency. In view of the current limitations regarding data scale and environmental complexity, subsequent work will expand the dataset to include a wider range of fruits and vegetables, and further study the effects of various environmental factors (such as temperature, humidity, and microbial contamination) on freshness detection. Collaborations with experts in agriculture, horticulture, and quality monitoring will be pursued to further refine data collection protocols, sample annotation, and evaluation criteria, thereby enhancing both data quality and model interpretability. Establishing an online learning mechanism for real-time model updates and adaptive adjustments is also a critical future direction, which will enable the model to better cope with evolving environmental and data distribution changes, thereby enhancing long-term stability and practicality in fruit and vegetable freshness monitoring and contributing to the development of a sustainable food industry.

## Figures and Tables

**Table 1:** Freshness grading of fruit images.

Freshness Level	Surface Characteristics
Good	No surface damage; full and vibrant color
Bad	Extensive damage or rotting with mold presence

**Table 2:** Data augmentation techniques and contributions.

Augmentation Technique	Description	Approximate Contribution to Sample Count (%)
Original Images	Unaltered raw images	100
Rotation (±30°)	Random rotation within ±30 degrees	80
Horizontal Flipping	Horizontal axis flip	80
Vertical Flipping	Vertical axis flip	50
Random Cropping	Random crops preserving object	60
Brightness and Contrast Adjustment	Simulated lighting variations	70
Gaussian Noise	Noise added for robustness	40

**Table 3:** ResNet-101 architecture.

Layer Name	Output Size	101-Layer
Conv1	112 × 112	7 × 7.64, Stride2
		3 × 3 max pool, Stride2
Conv2	56 × 56	$\left[\begin{array}{c} 1\times 1.64\\ 3\times 3.64\\ 1\times 1.256 \end{array}\right]\times$3
Conv3	28 × 28	$\left[\begin{array}{c} 1\times 1.128\\ 3\times 3.128\\ 1\times 1.512 \end{array}\right]\times$4
Conv4	14 × 14	$\left[\begin{array}{c} 1\times 1.256\\ 3\times 3.256\\ 1\times 1.1024 \end{array}\right]\times$24
Conv5	7 × 7	$\left[\begin{array}{c} 1\times 1.512\\ 3\times 3.512\\ 1\times 1.2048 \end{array}\right]\times 3$
	1 × 1	average pool, 1000-D fc, softmax
FLOPs		$7.6\;\times \;10^{9}$

**Table 4:** Training hyperparameters.

Hyperparameter	Value/Setting
Epoch Number	100
Batch Size	32
Initial Learning Rate	1 × 10^−4^
Learning Rate Scheduler	Cosine Annealing
Optimizer Type	Adam
Loss Function	Cross-Entropy Loss
Early Stopping	Enabled (patience = 5–10 epochs)
Hardware Platform	NVIDIA GTX 4060, Intel i9-13900HX, 16 GB RAM

**Table 5:** Experimental hardware configuration.

GPU	GTX4060
Central Processing Unit (CPU)	13th Gen Intel^®^ Core™ i9-13900HX × 32
Memory	16 GB RAM
Operating System (OS)	Ubuntu 22.04.5 LTS

**Table 6:** Dataset split and sample distribution.

Fruit Type	Freshness Class	Total Samples	Training Set (80%)	Validation Set (20%)
Apple	Good	620	496	124
Apple	Bad	396	317	79
Banana	Good	548	439	109
Banana	Bad	288	230	58
Orange	Good	543	459	114
Orange	Bad	351	281	70

**Table 7:** Comparative evaluation of overlap rate and false detection rate.

Model	OR (%)	False Detection Rate (%)
Resnet-101	83.6	6.4
Resnet-101 + Non-local Attention	91.8	3.2

**Table 8:** Inference time and parameter comparison.

Model	Average Inference Time (ms)	Parameter Count (M)
VGG-16	16.2	138
Resnet-50	12.8	25.6
Resnet-101	14.5	44.5
Resnet-101 + Non-local Attention	15.7	46.8

## Data Availability

The original contributions presented in this study are included in the article. Further inquiries can be directed to the corresponding author.
